# Photocytotoxic efficacy of sulphonated species of aluminium phthalocyanine against cell monolayers, multicellular spheroids and in vivo tumours.

**DOI:** 10.1038/bjc.1991.408

**Published:** 1991-11

**Authors:** W. S. Chan, C. M. West, J. V. Moore, I. R. Hart

**Affiliations:** Biology of Metastasis Laboratory, Imperial Cancer Research Fund, London, UK.

## Abstract

The problem of relying solely on in vitro data to predict photosensitiser efficacy was demonstrated by examining the uptake and the ability to mediate photocytotoxicity of mono-, di-, tri- and tetra-sulphonated species of chloroaluminium phthalocyanine (AlS1-4Pc) in monolayer cultures of murine Colo 26 cells and in both monolayer and spheroid cultures of human WiDr cells. Cells treated in vitro, whether in monolayer or as spheroids, with the less sulphonated derivatives, AlS1Pc and AlS2Pc, were more susceptible to photocytotoxicity than those treated with AlS3Pc, cells treated with AlS4Pc were even less susceptibile to the cytotoxic effects of light irradiation. Generally these results mirrored the cellular uptake in vitro. When WiDr spheroids were increased in size from 250 microns to 500 microns there was a reduction in uptake of AlS1Pc and AlS2Pc which was reflected by the decreased sensitivity of the larger spheroids to the effects of light irradiation. AlS1Pc had no effect against Colo 26 cells growing as s.c. tumours in syngeneic BALB/c mice; whereas AlS3Pc, AlS2Pc and AlS4Pc produced significant reductions in tumour weights 5 days post laser light irradiation. Of these, AlS2Pc had the most dramatic effect on the colony forming efficiency of tumour cells recovered 24 h after PDT. While, despite their effects on tumour size, AlS3Pc and AlS4Pc scarcely affected the subsequent viability of cells from dissociated tumours. Thus the in vitro efficacy of the sulphonated species of phthalocyanines is not necessarily predictive of their in vivo effectiveness.


Br. J. Cancer (1991), 64, 827-832 © Macmillan Press Ltd., 1991

### Photocytotoxic efficacy of sulphonated species of aluminium phthalocyanine against cell monolayers, multicellular spheroids and *in vivo* tumours

W.S. Chan[1,2], C.M.L. West*, J.V. Moore* & I.R. Hart[1]

[1]*Biology of Metastasis Laboratory, Imperial Cancer Research Fund, Lincoln's Inn Fields, London WC2A 3PX;* [2]*Radiobiology Department, Paterson Institute for Cancer Research, Christie Hospital and Holt Radium Institute, Wilmslow Road, Manchester M20 9BX, UK.*

**Summary** The problem of relying solely on *in vitro* data to predict photosensitiser efficacy was demonstrated by examining the uptake and the ability to mediate photocytotoxicity of mono-, di-, tri- and tetra-suphonated species of chloroaluminium phthalocyanine ($AlS_{1-4}Pc$) in monolayer cultures of murine Colo 26 cells and in both monolayer and spheroid cultures of human WiDr cells. Cells treated *in vitro*, whether in monolayer or as spheroids, with the less sulphonated derivatives, $AlS_1Pc$ and $AlS_2Pc$, were more susceptible to photocytotoxicity than those treated with $AlS_3Pc$, cells treated with $AlS_4Pc$ were even less susceptible to the cytotoxic effects of light irradiation. Generally these results mirrored the cellular uptake *in vitro*. When WiDr spheroids were increased in size from 250 µm to 500 µm there was a reduction in uptake of $AlS_1Pc$ and $AlS_2Pc$ which was reflected by the decreased sensitivity of the larger spheroids to the effects of light irradiation. $AlS_1Pc$ had no effect against Colo 26 cells growing as s.c. tumours in syngeneic BALB/c mice; whereas $AlS_3Pc$, $AlS_2Pc$ and $AlS_4Pc$ produced significant reductions in tumour weights 5 days post laser light irradiation. Of these, $AlS_2Pc$ had the most dramatic effect on the colony forming efficiency of tumour cells recovered 24 h after PDT. While, despite their effects on tumour size, $AlS_3Pc$ and $AlS_4Pc$ scarcely affected the subsequent viability of cells from dissociated tumours. Thus the *in vitro* efficacy of the sulphonated species of phthalocyanines is not necessarily predictive of their *in vivo* effectiveness.

The metallophthalocyanines (MPc) are good candidates for photosensitisers which may prove effective in the PDT of cancer (Spikes, 1986; Ben-Hur, 1987; van Lier *et al.*, 1987). One of the major advantages of the MPc is that this group of compounds absorbs light strongly in the red region (Q band – 670 nm); a region of the spectrum which permits good tissue penetration. Generally, the active MPc contain central diamagnetic metal ions, such as aluminium, gallium, tin and zinc (Brasseur *et al.*, 1985; Ben-Hur & Rosenthal, 1986; Brasseur *et al.*, 1987; Chan *et al.*, 1987*a*, 1988; Brasseur *et al.*, 1988; Paquette *et al.*, 1988) while sulphonation of the benzene rings of the macrocycle leads to solubility in water which facilitates administration to animals. One such compound, AlSPc, has been shown by ourselves (Chan *et al.*, 1986, 1988) and others (Sandeman *et al.*, 1987; Tralau *et al.*, 1987*a*; Nelson *et al.*, 1988) to be a particularly promising agent for PDT. This substance possesses good tumour-localising capacity (Tralau *et al.*, 1987*a*, 1987*b*; Chan *et al.*, 1988, 1989) and causes substantial damage to a range of tumours of diverse histological origin (Sandeman *et al.*, 1987; Tralau *et al.*, 1987*a*; Chan *et al.*, 1988; Nelson *et al.*, 1988) with prolongation of survival times in treated animals (Chan *et al.*, 1987*b*). Though AlSPc may induce a mild immunosuppression in mice (Marshall *et al.*, 1989) it produces much less skin photosensitivity than occurs subsequent to HpD or PII administration which is the current clinical regimen for PDT (Tralau *et al.*, 1989). AlSPc, as originally used by us (Chan *et al.*, 1986), was found to consist of a large number of isomers of species with varying degrees of sulphonation. The uptake and distribution of $AlS_nPc$ by tumour cells both *in vitro* and *in vivo* was found to be affected quite profoundly by the degree of sulphonation (Chan *et al.*, 1990). In this paper we have sought to determine whether these differences in uptake and distribution could affect efficacy as a consequence of the balance between dye uptake and penetration, by examining the effect of the variously sulphonated species on multicellular spheroids of differing sizes and on tumours *in vivo*.

Correspondence: I.R. Hart.
*Present address: MRC Radiobiology Unit, Chilton, Didcot, Oxon OX11 0RD, UK.
Received 20 May 1991; and in revised form 1 July 1991.
Abbreviations used: $AlS_nPc$, chloroaluminium sulphonated phthalocyanine with n sulphonate groups; AlSPc, chloroaluminium sulphonated phthalocyanine a mixture of mono- to tetra-sulphonated derivatives. BME, Eagle's basal medium; CFE, colony forming efficiency; DMF, dimethyl formamide; FCS, foetal calf serum; HBSS, Hank's balanced salt solution; HpD, haematoporphyrin derivative; MPc, metallophthalocyanines; PII, Photofrin II; PBS, phosphate buffered saline; Pc, phthalocyanine(s); PDT, photodynamic therapy.

### Materials and methods

### *Photosensitiser preparation*

AlSPc was obtained from Ciba-Geigy Dyestuffs and Chemicals (Basel, Switzerland). This material was a complex mixture of mono- to tetrasulphonated derivatives with, according to the supplier, an average of three sulphonate groups. $AlS_1Pc$, $AlS_2Pc$ and $AlS_3Pc$, individual chloroaluminium mono-, di- and tri-sulphonated derivatives, were bought from Porphyrin Products (Logan, Utah). $AlS_4Pc$, chloraluminium tetrasulphonated Pc, was prepared by the condensation of aluminium trichloride with sulphophthalic acid as detailed (Weber & Busch, 1965). These various derivatives were shown by spectroscopy and high pressure liquid chromatography analysis to be $>90\%$ pure though possibly containing numerous isomers (Chan *et al.*, 1990). The various dyes were dissolved in PBS except the water-insoluble $AlS_1Pc$ which was dissolved in 40% (v/v) ethanol:PBS for *in vivo* studies or in DMF for *in vitro* studies. The procedures of preparation and quantitation of the dye solutions have been detailed previously (Chan *et al.*, 1990). Since the molecular weights of the individual derivatives varied, equal molarities of these derivatives were compared for biological effects.

### *Tumour cells*

Colo 26 cells from a murine colorectal carcinoma, syngeneic to BALB/c mice, were passaged routinely in E4 growth medium containing 10% FCS (Chan *et al.*, 1987*b*). These cells were used both for *in vivo* and *in vitro* experiments.

WiDr cells from a human colon adenocarcinoma were passaged routinely in BME medium supplemented with 10% FCS (West, 1989). Multicellular spheroids of these cells were prepared by growing cultures in Petri dishes on a 1% agar base. Five days later the spheroid-containing culture supernatant was filtered to obtain homogeneously sized spheroids and the cells were grown in spinner flasks to the appropriate aggregate size as detailed elsewhere (West, 1989).

*Effects of* in vitro *irradiation of dye-treated cells*

Colo 26 cells were plated, ($5 \times 10^4 - 5 \times 10^5$), into 55 mm Petri dishes containing 5 ml growth medium and cultured for 1–3 days before being re-fed with fresh medium containing the various Pc derivatives at a final concentration of 10 μM. Following incubation in the dye-containing medium for 24 h, cells were washed three times with PBS, trypsinised, washed and counted. Cell concentrations were then adjusted and plated into triplicate Petri dishes, at cell numbers which ranged from $10^2 - 10^6$, containing dye-free medium. Cultures were exposed to red light at doses ranging up to 2.16 J $cm^{-2}$ and then maintained in incubators for a further 10–12 days before being fixed in methanol and stained with Giemsa to examine for colony formation. Only colonies of around 50 cells or greater were counted and the CFE was determined by relating these to colony numbers on untreated control dishes (red light irradiation, 2.16 J $cm^{-2}$, but no Pc-treatment or Pc-treatment with no light irradiation) which were expressed as 100% survival.

For the various experiments reported here as well as the effects of irradiation time (light dose), the effects of Pc-dose (from 0–30 μM at a fixed light dose of 2.16 J $cm^{-2}$) and incubation period in the presence of Pc were also examined.

The red light source (600–700 nm) consisted of a bank of six fluorescent tubes filtered through a red gelatin filter. The emission spectra of this source has been documented elsewhere (Chan *et al.*, 1986). The intensity of light measured at the cells' location was $1.2 \pm 0.2$ mW $cm^{-2}$ as determined by a Coherent Power Meter, Model 212 (Coherent Ltd., Cambridge, UK). These readings conflict with the value of 0.71 W $m^{-2}$ previously reported by our group (Chan *et al.*, 1986; 1987*a*) but we have established that the present figures are correct and the previous values arose as a consequence of a faulty power meter.

*Effect of $AlS_nPc$ plus light on WiDr cells growing in monolayer and as spheroids*

The procedure used to establish $AlS_nPc$-mediated cytotoxicity against WiDr cultures was similar to that described previously for PII (West, 1989). Briefly WiDr cells, in monolayer or as spheroids of 250 μm or 500 μm diameter, were incubated in growth medium containing a final concentration of 10 μM Pc derivative in spinner flasks for 24 h. Cultures were rinsed twice in PBS and disaggregated into single cell suspensions by trypsinisation. Cells were irradiated as single cell suspensions, $3-5 \times 10^5$ cells in 1 ml Hepes-buffered BME in 35 mm Petri dishes. Following irradiation with varying light doses up to a maximum of 15 J $cm^{-2}$ cells were then diluted to appropriate concentrations and plated into 60 mm Petri dishes containing BME. Colony counts were performed 20–22 days later and CFE was calculated as related to control cells (AlSnPc exposed monolayer/spheroid cultures but no light irradiation).

In these experiments red-light was derived from a copper vapour pumped dye laser (Oxford Laser, Oxford, UK) tuned to 675 nm (laser dye, Oxazine 720). Light was delivered via a 1 mm diameter quartz fibre which projected downward (West *et al.*, 1990) to produce an even light fluence (20 mW $cm^{-2}$) at the cell level as determined by a thermopile (Laser Instrumentation, Basingstoke, UK).

In vitro *Pc uptake*

The techniques used to determine this aspect of cell behaviour have been described in detail elsewhere (Chan *et al.*, 1990). Briefly, WiDr cells in the exponential growth phase, either as monolayers or spheriods, were exposed to 10 μM Pc-derivative for up to 48 h and cells were then dispersed by trypsinisation. Cell numbers were determined by Coulter counter and $10^6$ cells were centrifuged to form pellets. The cell pellets were digested in 0.1 M NaOH (0.1 M NaOH/ethanol for $AlS_1Pc$) and Pc content was determined by fluorospectrophotometry at the excitation/emission wavelengths appropriate for the specific sulphonated derivatives, as detailed previously (Chan *et al.*, 1990).

*PDT of Colo 26 tumours*

The different Pc-derivatives were used to sensitise BALB/c mice bearing s.c. Colo 26 tumours which were treated subsequently with laser light irradiation as described previously (Chan *et al.*, 1987*b*). Tumours were produced by injecting $10^6$ cells s.c. into the flank region of female BALB/c mice (12–14 weeks old, obtained from the Imperial Cancer Research Fund Animal Breeding Unit, Clare Hall, Herts, UK). When tumours were approximately 5–7 mm diameter the mice received an injection of 0.1 ml of a 1.134 mM solution of one of the Pc-derivatives via the lateral tail vein. Twenty-four hours later PDT was performed using a copper vapour pumped dye laser (675 nm, power 50 mW). Mice were under anaesthesia during PDT (Chan *et al.*, 1987*b*). Light was delivered to the centre of the tumour via a 0.2 mm diameter quartz fibre (100 J/tumour) as detailed previously (Chan *et al.*, 1987*b*). The tumour response to PDT was evaluated by (a) measuring tumour weight 5 days after light irradiation and (b) by determination of the number of clonogenic cells recovered from disaggregated tumour 24 h after PDT. Controls were obtained from mice where laser irradiation of tumours had been performed, but the animals had not received any dye injection; values obtained from this group were expressed as 100%. The disaggregation of tumours was achieved by incubating minced tumour in 0.02% collagenase (Type 1; Sigma Chemical Company, Poole, UK) and 0.01% DNAase (Type 1; Sigma) for 2–2½ h at 37°C with continuous stirring. Cells were washed with PBS, pelleted and resuspended in growth medium and viability was determined on an aliquot by trypan blue exclusion. From 100 to 5,000 viable cells were plated into 60 mm Petri dishes (3–6 dishes per cell dose) and CFE was calculated 10–14 days later as described above.

### Results

*Photosensitising activity of sulphonated Pc-derivatives against Colo 26 and WiDr cells* in vitro

Results from between 3–6 independent experiments are summarised in Figure 1. Increasing sulphonation was associated with diminished photocytoxocity such that $AlS_3Pc$ and $AlS_4Pc$ were unable to reduce the surviving fraction of treated Colo 26 cells to any significant extent (Figure 1a). By contrast, at light doses of 2.16 J $cm^{-2}$, $AlS_1Pc$ and $AlS_2Pc$ reduced the surviving fraction of Colo 26 cells by greater than 99%. The relative lack of efficacy of $AlS_3Pc$ and $AlS_4Pc$ was maintained even at high concentrations of the two species (Figure 1b). Despite the fact that the AlSPc mixture was claimed to have an average of three sulphonate groups, its behaviour most closely resembled that of the di-sulphonate species (Figures 1a–1c).

Although the range of light doses used against the WiDr cells, both in monolayer culture and as spheroids of different size, were greater than those used in the experiments with Colo 26 cells, a similar pattern of potency was observed (Figure 2). Thus the photokilling capacity of $AlS_1Pc > AlS_2Pc > AlS_3Pc > AlS_4Pc$ whether cells were grown as monolayer cultures (Figure 2a) or as spheroids (Figure 2b,2c).

The size of the spheroids used in these experiments (250 μm or 500 μm diameter) had an observable effect upon the response of treated cells to light irradiation (Figures 2b

**Figure 1** Clonogenic capacity of Colo 26 cells treated in monolayer with sulphonated Pc-derivatives. Colony forming efficiency of treated cells was examined as a function of **a**, light dose (10 μM $AlS_nPc$ in culture medium for 24 h prior to irradiation) **b**, dye concentration (irradiation with 2.16 J cm$^{-2}$ red light after 24 h exposure to $AlS_nPc$) and **c**, exposure time to dye (constant 10 μM $AlS_nPc$ and 2.16 J cm$^{-2}$ red light). O, $AlS_1Pc$; ▲, $AlS_2Pc$; □, $AlS_3Pc$; ■, $AlS_4Pc$; Δ, AlSPc. Vertical bars, standard errors (s.e.). Data points derived from triplicate dishes in a minimum of three independent experiments.

**Figure 2** Clonogenic capacity of WiDr cells after exposure to 10 μM of individual Pc derivative then graded red light irradiation **a**, monolayer, **b**, 250 μm diameter or **c**, 500 μm diameter spheroids, O, $AlS_1Pc$; ▲, $AlS_2Pc$; □, $AlS_3Pc$; ■, $AlS_4Pc$. Results presented are derived from three independent experiments; vertical bars, s.e.

and 2c). Furthermore there was a fairly marked difference between the response of cells in monolayer (Figure 2a) and as spheroids (Figures 2b and 2c). Thus the production of equivalent cytotoxicities by $AlS_1Pc$ and $AlS_2Pc$ required higher light doses in spheroids that it did in monolayer cells and it appeared that $AlS_1Pc$ and $AlS_2Pc$ were more effective against cells in the 250 μm aggregates than in the 500 μm diameter spheroids. These differences were less marked for $AlS_3Pc$ and were not apparent for $AlS_4Pc$.

### *Cellular uptake of Pc derivatives* in vitro

The kinetics of uptake of the various sulphonated species of $AlS_nPc$ by WiDr cells growing in monolayer or as different sized spheroids are presented in Figure 3. The derived uptake curves are very similar to those already established for Colo 26 cells (Chan *et al.*, 1990) and indicate that the relative amount of each individual species taken up by the cells varies considerably with $AlS_1Pc > AlS_2Pc > AlS_4Pc \geq AlS_3Pc$. The qualitative and quantitative patterns of uptake by the spheroids of the various species were remarkably similar for both sizes of spheroids (Figures 3b and 3c). It was clear however that there was a noticeable decrease in the amount of $AlS_2Pc$ taken up by spheroids, and a smaller decrease in the amount of $AlS_1Pc$, as compared with that taken up by monolayer cells.

### *Anti-tumour efficacy of Pc derivatives in PDT*

The effect of PDT on s.c. located growing Colo 26 tumours is illustrated in Figure 4 where the weights of tumours from the various groups, 5 days after light irradiation, are presented. It is apparent that $AlS_1Pc$ had no effect upon tumour weight but that the use of $AlS_2Pc$, $AlS_3Pc$, $AlS_4Pc$ or AlSPc all induced significant reductions ($P < 0.001$) in tumour size.

Apart from the changes in total tumour weights there were also changes in the clonogenic capacity of cells recovered from treated tumours 24 h after the application of PDT (Figure 5). In this assay it can be seen that $AlS_2Pc$ and AlSPc were by far the most potent dyes; reducing the surviving fraction to less than 1% of that obtained from control (untreated) tumours. Because $AlS_1Pc$ had no effect on tu-

**Figure 3** Kinetics of cellular uptake *in vitro* of sulphonated Pc by WiDr cells **a**, in monolayer, **b**, as 250 μm spheroids or **c**, as 500 μm spheroids. O, $AlS_1Pc$; ▲, $AlS_2Pc$; □, $AlS_3Pc$; ■, $AlS_4Pc$. Pc concentration in the medium is 10 μM. Bars, standard deviations (s.d.). Earliest time point 1 h after addition of Pc.

**Figure 4** Colo 26 tumour weights 5 days after PDT. Tumour-bearing mice were alloted to one of six groups: mice received **a**, PBS; **b**, $AlS_1Pc$; **c**, $AlS_2Pc$; **d**, $AlS_3Pc$; **e**, $AlS_4Pc$; **f**, AlSPc. Individual species of dye (0.1 ml of 1.135 mM) were injected i.v. via tail vein 24 h prior to PDT. ▨, anaesthesia but no laser irradiation; ▤, laser light irradiation. Numbers at base of columns represent individual tumours per group. Bars, s.d. of mean tumour weights.

**Figure 5** Clonogenic capacity of Colo 26 cells recovered from tumours after the same PDT treatment as described in Figure 4. Cells recovered from tumours of animals which had received, **a**, $AlS_2Pc$; **b**, $AlS_3Pc$; **c**, $AlS_4Pc$ or **d**, AlSPc. Number of colonies per gram of tumour obtained from control mice (no dye injection but with laser irradiation) are expressed as 100% survival. Bars, s.e.

mour weight no attempt was made to evaluate the effect of this species on clonogenic capacity.

### Discussion

The results presented in this study show that the degree of sulphonation of chloroaluminium phthalocyanine can have a profound effect upon light-induced cytotoxicity *in vitro* and *in vivo*. Thus against cells and spheroids in tissue culture it is the least sulphonated species which are the most phototoxic (Figures 1 and 2). Conversely when Colo 26 tumours *in vivo* were subjected to PDT protocols (Chan *et al.*, 1987*b*) it was apparent that $AlS_1Pc$, a highly potent photosensitiser *in vitro*, had virtually no cytotoxic capacity (Figure 4). These results appear to be due, partially at least, to the relative differences in uptake that exist between cells in tissue culture or *in vivo* (Chan *et al.*, 1990). Since the degree of sulphonation is correlated inversely to the lipid/water partition coefficient (Berg *et al.*, 1989*a*) an increase in the number of sulphonation groups would lead to a decrease in Pc lipophilicity. The greater the partition coefficient, the higher the concentration of the drug in the membrane and the faster the rate of diffusion into the cell (Benet & Sheiner, 1985). In accord with this possibility the less sulphonated Pc are taken up to a greater extent, and at a faster rate, than the more sulphonated Pc in tissue culture cells (see Chan *et al.*, 1990; and Figure 3). This *in vitro* correlation exists with WiDr cells whether they are growing in monolayer culture or as spheroids of two different sizes. $AlS_1Pc$ and $AlS_2Pc$ are taken up to lesser extent by cells in spheroids than by cells in monolayer culture (Figure 3) and this reduced accumulation in spheroids is reflected in the increased light dose required to achieve comparable levels of cell killing (Figure 2). It appears from our experiments in tissue culture that observed cytotoxicity mainly is a consequence of photosensitiser uptake where decreased sulphonation is correlated with increased phototoxic efficacy. While these data agree with those obtained with some other phthalocyanines, $GaS_nPc$ for example (Brasseur *et al.*, 1987), they are in conflict with others findings on $ZnS_nPc$ and even $AlS_nPc$ (Brasseur *et al.*, 1988; Berg *et al.*, 1989*b*). The reason for these discrepancies is not known but may relate to either differences in purity or isomer content of the photosensitising agents. Also, it has been proposed that disulphonated material where the two sulphonate groups are adjacent may be more effective than material where the sulphonate groups are on opposite sides of the molecule (Paquette *et al.*, 1988). Direct comparison of our results with others will require further work to characterise both the isomer content and the position of the sulphonate groups.

It had also been shown that the amount of PII, a more hydrophobic sensitiser (Berg *et al.*, 1989*a*), taken up by spheroids diminished markedly with increasing aggregate size, as did resultant phototoxicity (West, 1989). Thus the less hydrophilic Pc derivatives used in this study, $AlS_1Pc$ and $AlS_2Pc$, showed a reduction in accumulation in WiDr cells growing as spheroids relative to monolayer cells, and the differences for $AlS_2Pc$ were similar (Figure 3) to those obtained with PII (West, 1989). These findings suggest that the contrast in Pc-derivative accumulation and retention observed in the *in vitro* and the *in vivo* situation (Chan *et al.*, 1990) may be attributable partially to lower penetration into three-dimensional, as compared to two-dimensional, tumour foci by hydrophobic species. We have shown previously that Colo 26 growing s.c. in BALB/c mice accumulated photosensitiser to a greater extent as the degree of sulphonation increased so that $AlS_4Pc > AlS_3Pc > AlS_2Pc > AlS_1Pc$ (Chan *et al.*, 1990). We now show that $AlS_1Pc$ is ineffective in the PDT of s.c. tumours whereas the other Pc derivatives can reduce substantially the size of treated tumours (Figure 4) or the clonogenic capacity of component neoplastic cells (Figure 5). Others have found that the more hydrophilic, $AlS_3Pc$ and $AlS_4Pc$, derivatives localise mainly in the extracellular stromal compartment of the tumour (Peng *et al.*, 1990*a*, 1990*b*). This suggests that one possible reason for the relative inefficiency of $AlS_{3-4}Pc$ compared with $AlS_2Pc$ may be that, though the latter derivative is taken up to a lesser extent, what is accumulated is localised in an area where it is able to exert maximum effect. Other groups have also shown $AlS_2Pc$ to be a more effective photosensitiser than HpD (Canti *et al.*, 1990).

We have suggested that a possible explanation for the dearth of $AlS_1Pc$ in tumour tissue may be the efficient removal of this dye from the circulation by hepatic accumulation and retention (Chan *et al.*, 1990). The effects of the sulphonated species on tumour weight and clonogenic cells (Figures 4 and 5) show some discordancy. Thus $AlS_3Pc$ and $AlS_4Pc$ evoke significant reductions in tumour size (Figure 4) but have little effect on the CFE of cells from the disaggregated tumour (Figure 5). It may be that the gross weights of tumours provides an inaccurate assay relative to the CFE assay. Alternatively $AlS_3Pc$ and $AlS_4Pc$ have been stated to exert a greater effect against tumour vasculature (Henderson & Farrell, 1989; Peng *et al.*, 1990*c*) and their mode of cell killing may differ from that of the more potent $AlS_2Pc$. Henderson and Farrell (1989) claimed that an enriched fraction of the mono-sulphonated species was the most effective photosensitiser *in vivo* compared to the other sulphonated species. However since these workers were able to dissolve the dye in PBS, it is possible that the effect they observed might have been due to the presence of different isomers or additional sulphonated species (Henderson & Farrell, 1989). Such a possibility would explain the differences from our results.

Whatever the relative role or importance of this type of pharmacokinetics and intra-tumoural location of Pc-derivatives it is clear from our studies that a thorough understanding of not only the *in vitro* characteristics of Pc dyes but also an appreciation of their *in vivo* behaviour is required before selection of the optimal component for PDT can be made.

We wish to thank Drs M.M. Coombs and A. Creighton, Imperial Cancer Research Fund Laboratories, for their helpful comments.

### References

BENET, L.Z. & SHEINER, L.B. (1985). Pharmacokinetics: the dynamics of drug absorption, distribution, and elimination. In *The Pharmacological basis of Therapeutics*, Gilman, A.G., Goodman, L.S., Rall, T.W. & Maurad, F. (eds), pp. 3–34. MacMillan: New York.

BEN-HUR, E. & ROSENTHAL, I. (1986). Photosensitization of Chinese hamster cells by water-soluble phthalocyanines. *Photochem. Photobiol.*, **43**, 615.

BEN-HUR, E. (1987). Photochemistry and photobiology of phthalocyanines: new sensitizers for photodynamic therapy of cancer. In *From Photophysics to Photobiology*, Fave, A., Tyrell, R. & Cadet, J. (eds), pp. 407–420. Elsevier: New York.

BERG, K., BOMMER, J.C. & MOAN, J. (1989*a*). Evaluation of sulfonated aluminium phthalocyanines for use in photochemotherapy. Cellular uptake studies. *Cancer Lett.*, **44**, 7.

BERG, K., BOMMER, J.C. & MOAN, J. (1989*b*). Evaluation of sulfonated aluminium phthalocyanines for use in photochemotherapy. A study on the relative efficiencies of photoinactivation. *Photochem. Photobiol.*, **49**, 587.

BRASSEUR, N., ALI, H., AUTENRIETH, D., LANGLOIS, R. & VAN LIER, J.E. (1985). Biological activities of phthalocyanines – III. Photoinactivation of V-79 Chinese hamster cells by tetrasulfophthalocyanines. *Photochem. Photobiol.*, **42**, 515.

BRASSEUR, N., ALI, H., LANGLOIS, R. & VAN LIER, J.E. (1987). Biological activities of phthalocyanines – VII. Photoinactivation of V-79 Chinese hamster cells by selectively sulfonated gallium phthalocyanines. *Photochem. Photobiol.*, **45**, 739.

BRASSEUR, N., ALI, H., LANGLOIS, R. & VAN LIER, J.E. (1988). Biological activities of phthalocyanines – IX. Photosensitization of V-79 Chinese hamster cells and EMT-6 mouse mammary tumor by selectively sulfonated zinc phthalocyanines. *Photochem. Photobiol.*, **47**, 705.

CANTI, G., FRANCO, P., MARELLI, O., CUBEDDU, R., TARONI, P. & RAMPONI, R. (1990). Comparative study of the therapeutic effect of photoactivated hematoporphyrin derivative and aluminium disulfonated phthalocyanine on tumour bearing mice. *Cancer Lett.*, **53**, 123.

CHAN, W.-S., SVENSEN, R., PHILLIPS, D. & HART, I.R. (1986). Cell uptake, distribution and response to aluminium chlorosulphonated phthalocyanine, a potential anti-tumour photosensitizer. *Br. J. Cancer*, **53**, 255.

CHAN, W.-S., MARSHALL, J.F., SVENSEN, R., PHILLIPS, D. & HART, I.R. (1987*a*). Photosensitising activity of phthalocyanine dyes screened against tissue culture cells. *Photochem. Photobiol.*, **45**, 757.

CHAN, W.-S., MARSHALL, J.F. & HART, I.R. (1987*b*). Photodynamic therapy of a murine tumor following sensitisation with chloro aluminium sulfonated phthalocyanine. *Photochem. Photobiol.*, **46**, 867.

CHAN, W.-S., MARSHALL, J.F., LAM, G.Y.F. & HART, I.R. (1988). Tissue uptake, distribution, and potency of the photoactivatable dye chloroaluminium sulfonated phthalocyanine in mice bearing transplantable tumors. *Cancer Res.*, **48**, 3040.

CHAN, W.-S., MARSHALL, J.F. & HART, I.R. (1989). Effect of tumour location on selective uptake and retention of phthalocyanines. *Cancer Lett.*, **44**, 73.

CHAN, W.-S., MARSHALL, J.F., SVENSEN, R., BEDWELL, J. & HART, I.R. (1990). Effect of sulfonation on the cell and tissue distribution of the photosensitizer aluminium phthalocyanine. *Cancer Res.*, **50**, 4533.

HENDERSON, B.W. & FARRELL, G. (1989). Possible implications of vascular damage for tumor cell inactivation *in vivo*: comparison of different photosensitizers. *SPIE Proc.*, 1065.

MARSHALL, J.F., CHAN, W.-S. & HART, I.R. (1989). Effect of photodynamic therapy on anti-tumor immune defenses: comparison of the photosensitizers hematoporphyrin derivatives and chloro-aluminium sulfonated phthalocyanine. *Photochem. Photobiol.*, **49**, 627.

NELSON, J.S., LIAW, L.-H., ORENSTEIN, A., ROBERTS, W.G. & BERNS, M.W. (1988). Mechanism of tumor destruction following photodynamic therapy with hematoporphyrin derivative, chlorin, and phthalocyanine. *J. Natl Cancer Inst.*, **80**, 1599.

PAQUETTE, B., ALI, H., LANGLOIS, R. & VAN LIER, J.E. (1988). Biological activities of phthalocyanines – VIII. Cellular distribution in V-79 Chinese hamster cells and phototoxicity of selectively sulfonated aluminium phthalocyanines. *Photochem. Photobiol.*, **47**, 215.

PENG, Q., MOAN, J., FARRANTS, G., DANIELSEN, H.E. & RIMINGTON, C. (1990*a*). Localization of potent photosensitizers in human tumor LOX by means of laser scanning microscopy. *Cancer Lett.*, **53**, 129.

PENG, Q., NESLAND, J.M., MOAN, J., EVENSEN, J.E., KONGSHAUG, M. & RIMINGTON, C. (1990*b*). Localization of fluorescent Photofrin II and aluminium phthalocyanine tetrasulfonate in transplanted human malignant tumor LOX and normal tissues of nude mice using highly light-sensitive video intensification microscopy. *Int. J. Cancer*, **45**, 972.

PENG, Q., MOAN, J., NESLAND, J.M. & RIMINGTON, C. (1990*c*). Aluminium phthalocyanines with asymmetrical lower sulfonation and with symmetrical higher sulfonation: a comparison of localizing and photosensitizing mechanism in human tumor LOX xenografts. *Int. J. Cancer*, **46**, 719.

SANDEMAN, D.R., BRADFORD, R., BUXTON, P., BOWN, S.G. & THOMAS, D.G.T. (1987). Selective necrosis of malignant gliomas in mice using photodynamic therapy. *Br. J. Cancer*, **55**, 647.

SPIKES, J.D. (1986). Phthalocyanines as photosensitizers in biological systems and for the photodynamic therapy of tumors. *Photochem. Photobiol.*, **43**, 691.

TRALAU, C.J., MACROBERT, A.J., COLERIDGE-SMITH, P.D., BARR, H. & BOWN, S.G. (1987*a*). Photodynamic therapy with phthalocyanine sensitisation: quantitative studies in a transplantable rat fibrosarcoma. *Br. J. Cancer*, **55**, 389.

TRALAU, C.J., BARR, H., SANDEMAN, D.R., BARTON, T., LEWIN, M.R. & BOWN, S.G. (1987*b*). Aluminium sulfonated phthalocyanine distribution in rodent tumors of the colon, brain and pancreas. *Photochem. Photobiol.*, **46**, 777.

TRALAU, C.J., YOUNG, A.R., WALKER, N.P.J. & 4 others (1989). Mouse skin photosensitivity with dihaematoporphyrin ether (DHE) aluminium sulphonated phthalocyanine (AlSPc): a comparative study. *Photochem. Photobiol.*, **49**, 305.

VAN LIER, J.E., BRASSEUR, N., PAQUETTE, B., WAGNER, J.R., ALI, H., LANGLOIS, R. & ROUSSEAU, J. (1987). Phthalocyanines as sensitizers for photodynamic therapy of cancer. In *Photosensitization: Molecular and Medical Aspects*, Moreno, G., Pottier, R.H. & Truscott, T.G. (eds), pp. 435. Springer Verlag: Berlin.

WEBER, J.H. & BUSCH, D.H. (1965). Complex derived from strong field ligands. XIX. Magnetic properties of transition metal derivatives of 4, 4′, 4″, 4‴-tetrasulfophthalocyanine. *Inorg. Chem.*, **4**, 469.

WEST, C.M.L. (1989). Size-dependent resistance of human tumour spheroids to photodynamic treatment. *Br. J. Cancer*, **59**, 510.

WEST, C.M.L., WEST, D.C., KUMAR, S. & MOORE, J.V. (1990). A comparison of the sensitivity to photodynamic treatment of endothelial and tumour cells in different proliferative states. *Int. . Radiat. Biol.*, **58**, 145.


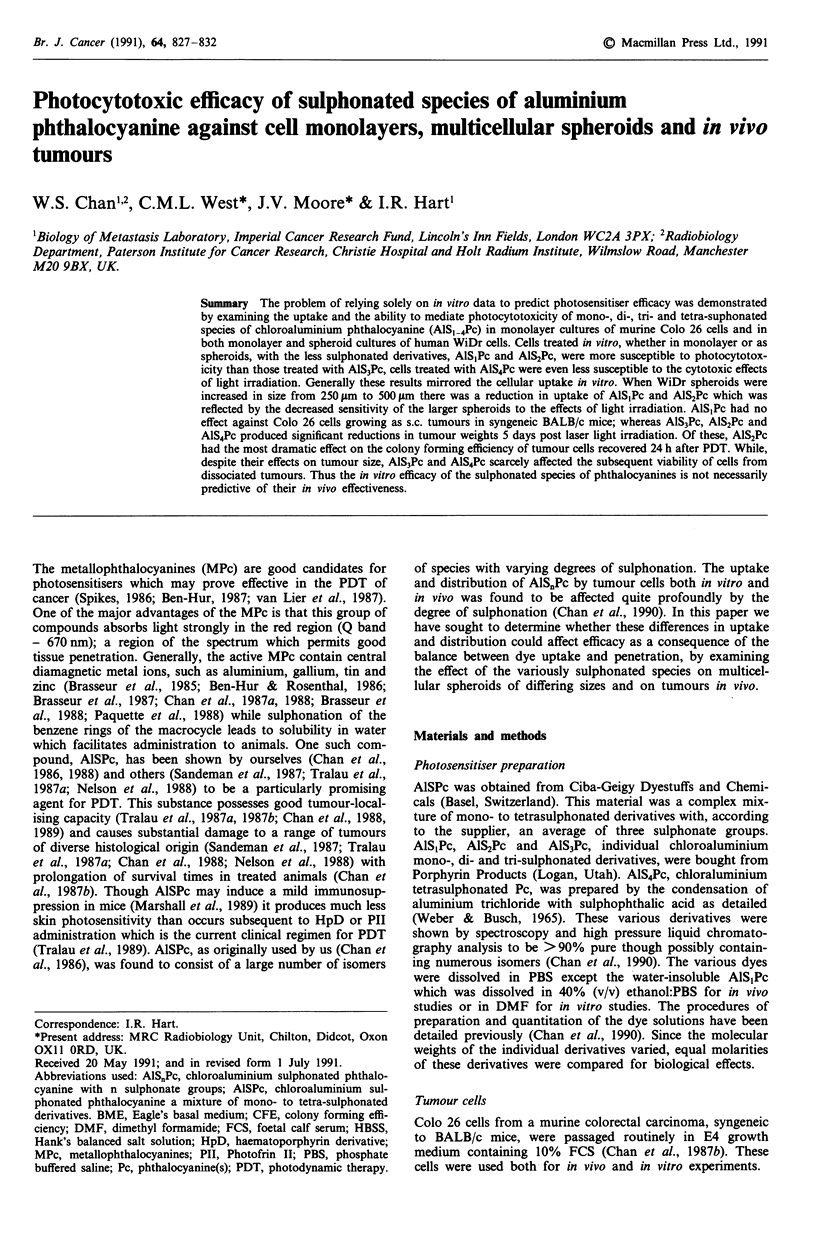

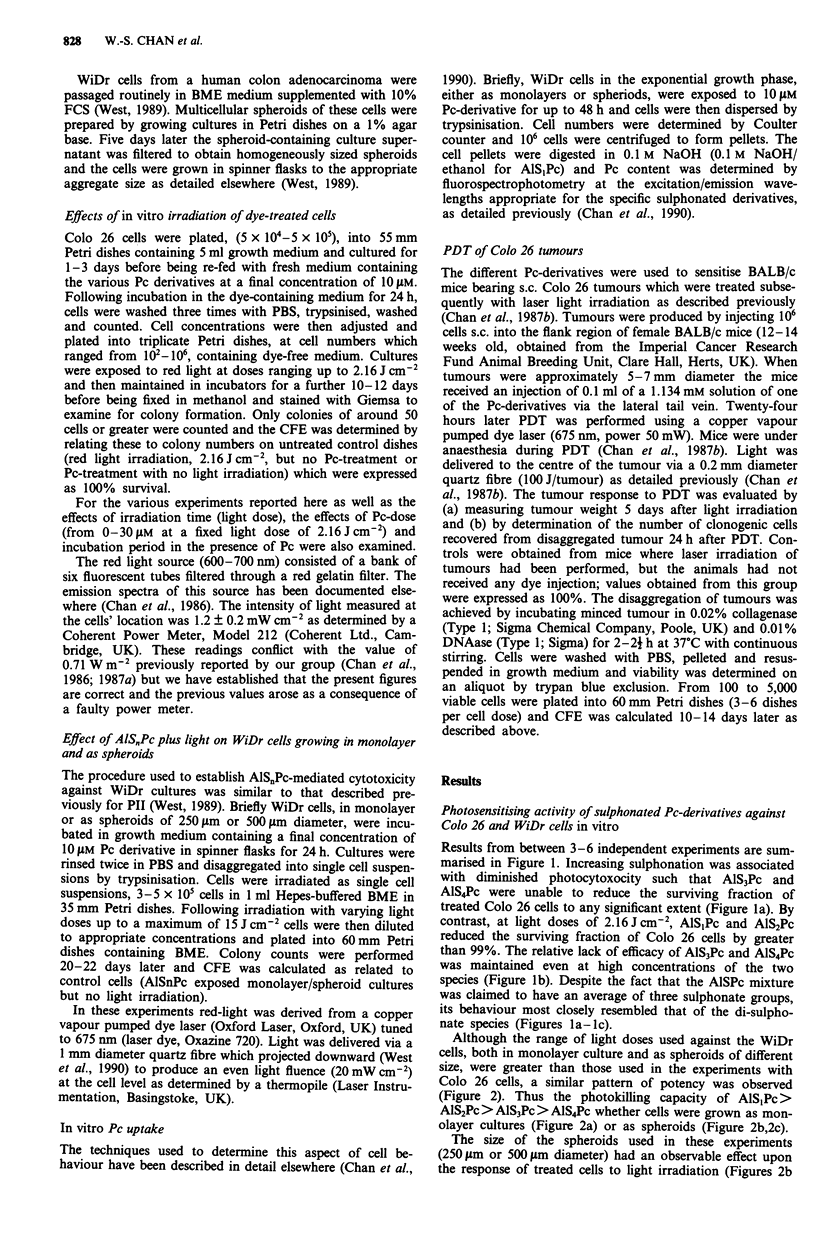

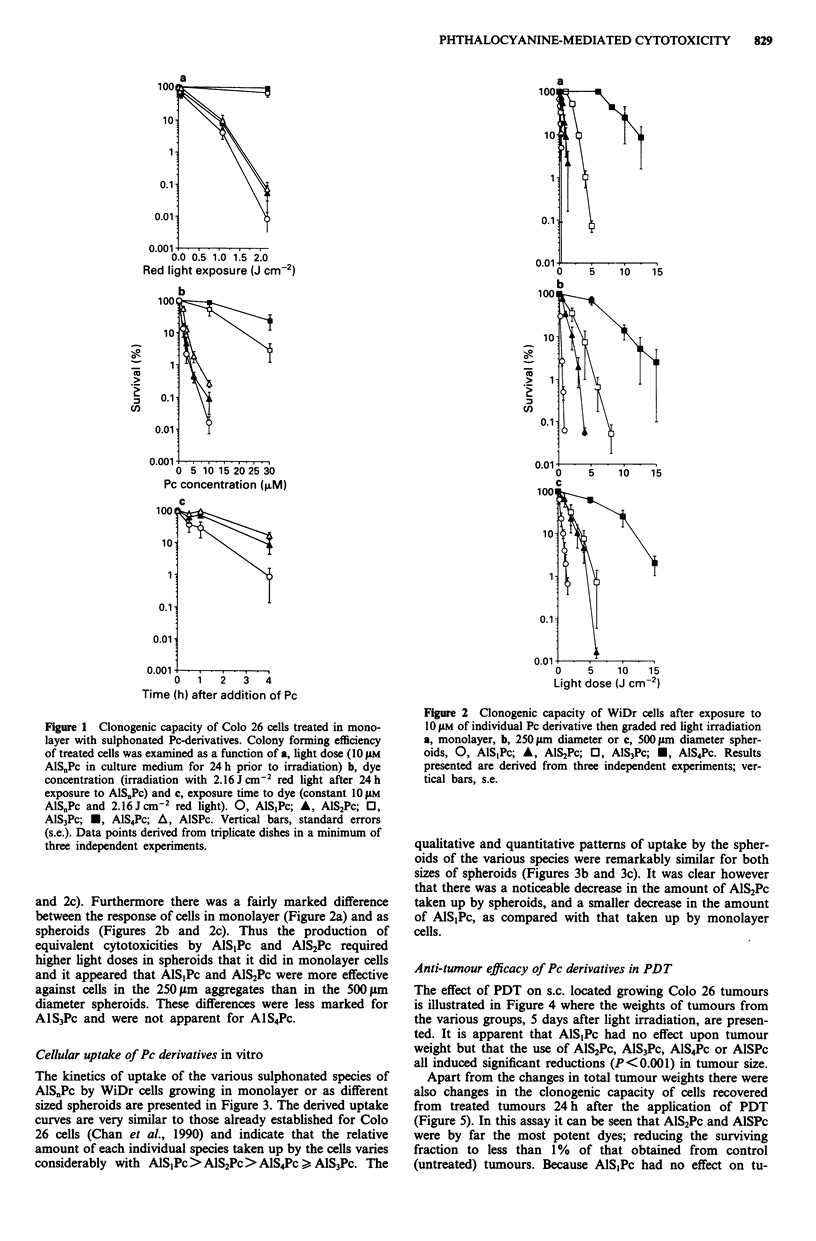

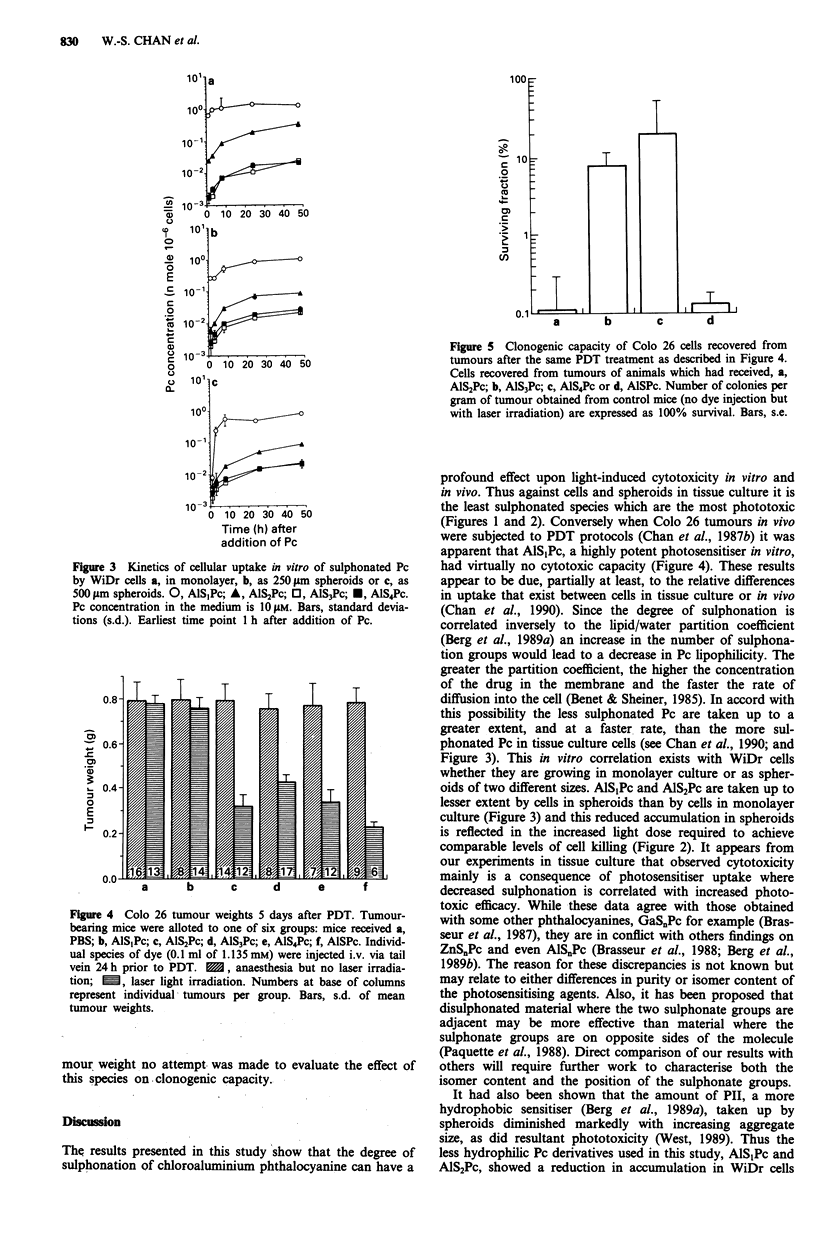

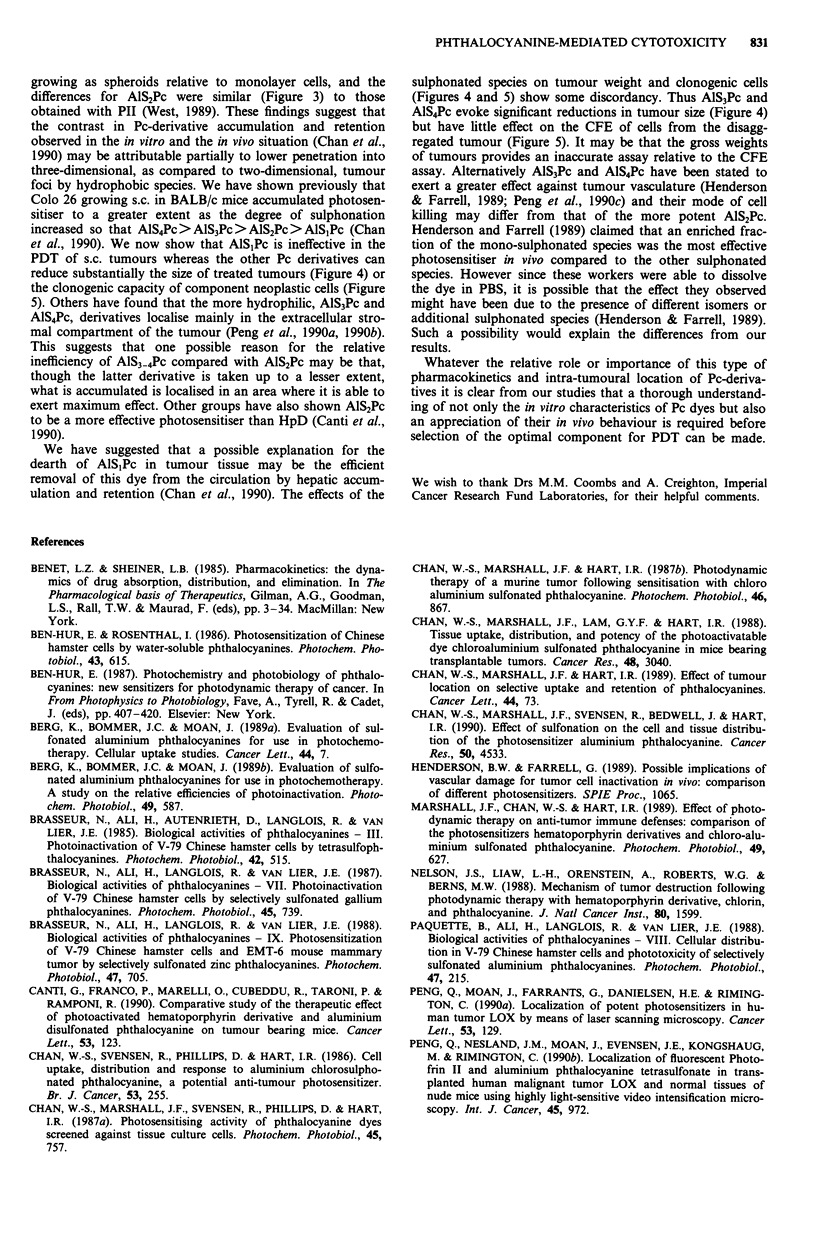

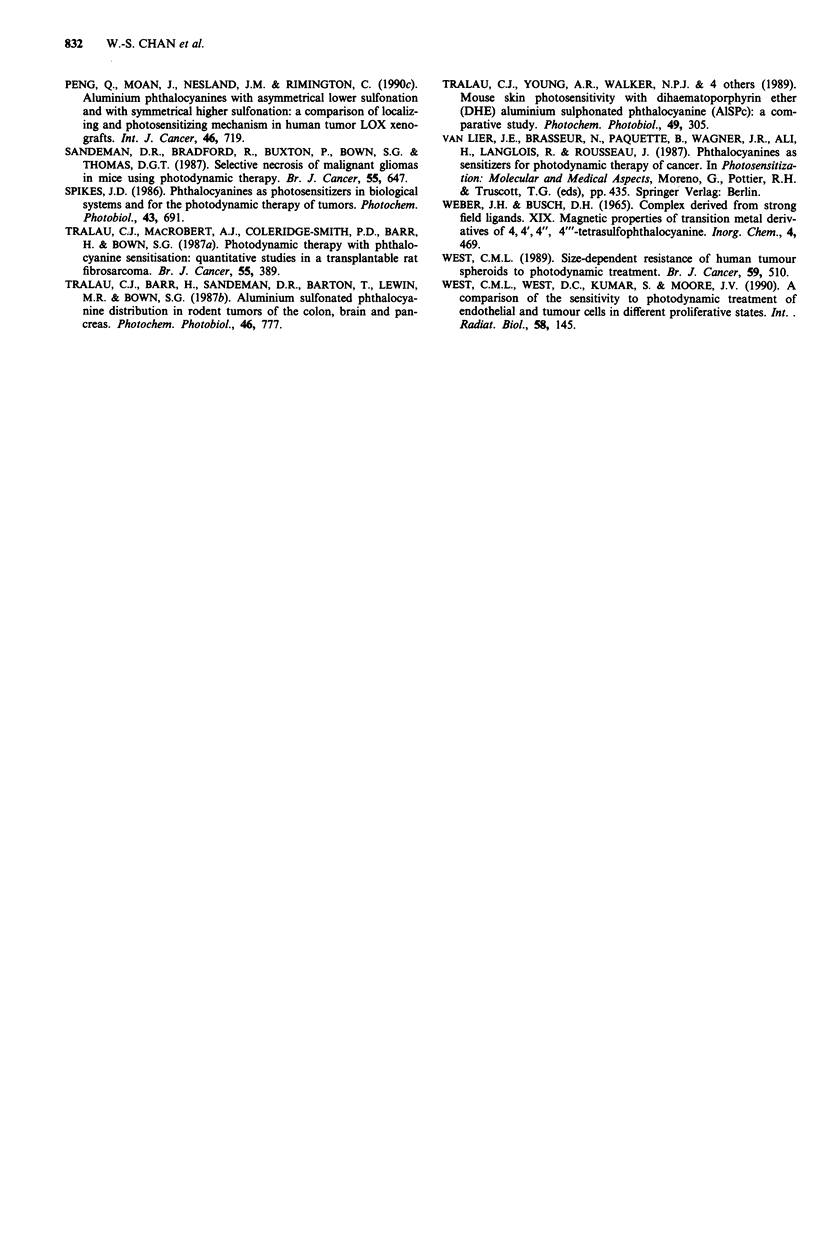


## References

[OCR_00674] Ben-Hur E., Rosenthal I. (1986). Photosensitization of Chinese hamster cells by water-soluble phthalocyanines.. Photochem Photobiol.

[OCR_00690] Berg K., Bommer J. C., Moan J. (1989). Evaluation of sulfonated aluminum phthalocyanines for use in photochemotherapy. A study on the relative efficiencies of photoinactivation.. Photochem Photobiol.

[OCR_00685] Berg K., Bommer J. C., Moan J. (1989). Evaluation of sulfonated aluminum phthalocyanines for use in photochemotherapy. Cellular uptake studies.. Cancer Lett.

[OCR_00696] Brasseur N., Ali H., Autenrieth D., Langlois R., van Lier J. E. (1985). Biological activities of phthalocyanines--III. Photoinactivation of V-79 Chinese hamster cells by tetrasulfophthalocyanines.. Photochem Photobiol.

[OCR_00708] Brasseur N., Ali H., Langlois R., van Lier J. E. (1988). Biological activities of phthalocyanines--IX. Photosensitization of V-79 Chinese hamster cells and EMT-6 mouse mammary tumor by selectively sulfonated zinc phthalocyanines.. Photochem Photobiol.

[OCR_00702] Brasseur N., Ali H., Langlois R., van Lier J. E. (1987). Biological activities of phthalocyanines--VII. Photoinactivation of V-79 Chinese hamster cells by selectively sulfonated gallium phthalocyanines.. Photochem Photobiol.

[OCR_00715] Canti G., Franco P., Marelli O., Cubeddu R., Taroni P., Ramponi R. (1990). Comparative study of the therapeutic effect of photoactivated hematoporphyrin derivative and aluminum disulfonated phthalocyanines on tumor bearing mice.. Cancer Lett.

[OCR_00746] Chan W. S., Marshall J. F., Hart I. R. (1989). Effect of tumour location on selective uptake and retention of phthalocyanines.. Cancer Lett.

[OCR_00734] Chan W. S., Marshall J. F., Hart I. R. (1987). Photodynamic therapy of a murine tumor following sensitisation with chloro aluminum sulfonated phthalocyanine.. Photochem Photobiol.

[OCR_00740] Chan W. S., Marshall J. F., Lam G. Y., Hart I. R. (1988). Tissue uptake, distribution, and potency of the photoactivatable dye chloroaluminum sulfonated phthalocyanine in mice bearing transplantable tumors.. Cancer Res.

[OCR_00751] Chan W. S., Marshall J. F., Svensen R., Bedwell J., Hart I. R. (1990). Effect of sulfonation on the cell and tissue distribution of the photosensitizer aluminum phthalocyanine.. Cancer Res.

[OCR_00728] Chan W. S., Marshall J. F., Svensen R., Phillips D., Hart I. R. (1987). Photosensitising activity of phthalocyanine dyes screened against tissue culture cells.. Photochem Photobiol.

[OCR_00722] Chan W. S., Svensen R., Phillips D., Hart I. R. (1986). Cell uptake, distribution and response to aluminium chloro sulphonated phthalocyanine, a potential anti-tumour photosensitizer.. Br J Cancer.

[OCR_00762] Marshall J. F., Chan W. S., Hart I. R. (1989). Effect of photodynamic therapy on anti-tumor immune defenses: comparison of the photosensitizers hematoporphyrin derivative and chloro-aluminum sulfonated phthalocyanine.. Photochem Photobiol.

[OCR_00769] Nelson J. S., Liaw L. H., Orenstein A., Roberts W. G., Berns M. W. (1988). Mechanism of tumor destruction following photodynamic therapy with hematoporphyrin derivative, chlorin, and phthalocyanine.. J Natl Cancer Inst.

[OCR_00775] Paquette B., Ali H., Langlois R., van Lier J. E. (1988). Biological activities of phthalocyanines--VIII. Cellular distribution in V-79 Chinese hamster cells and phototoxicity of selectively sulfonated aluminum phthalocyanines.. Photochem Photobiol.

[OCR_00784] Peng Q., Moan J., Farrants G., Danielsen H. E., Rimington C. (1990). Localization of potent photosensitizers in human tumor LOX by means of laser scanning microscopy.. Cancer Lett.

[OCR_00798] Peng Q., Moan J., Nesland J. M., Rimington C. (1990). Aluminum phthalocyanines with asymmetrical lower sulfonation and with symmetrical higher sulfonation: a comparison of localizing and photosensitizing mechanism in human tumor LOX xenografts.. Int J Cancer.

[OCR_00788] Peng Q., Nesland J. M., Moan J., Evensen J. F., Kongshaug M., Rimington C. (1990). Localization of fluorescent Photofrin II and aluminum phthalocyanine tetrasulfonate in transplanted human malignant tumor LOX and normal tissues of nude mice using highly light-sensitive video intensification microscopy.. Int J Cancer.

[OCR_00805] Sandeman D. R., Bradford R., Buxton P., Bown S. G., Thomas D. G. (1987). Selective necrosis of malignant gliomas in mice using photodynamic therapy.. Br J Cancer.

[OCR_00810] Spikes J. D. (1986). Phthalocyanines as photosensitizers in biological systems and for the photodynamic therapy of tumors.. Photochem Photobiol.

[OCR_00821] Tralau C. J., Barr H., Sandeman D. R., Barton T., Lewin M. R., Bown S. G. (1987). Aluminum sulfonated phthalocyanine distribution in rodent tumors of the colon, brain and pancreas.. Photochem Photobiol.

[OCR_00815] Tralau C. J., MacRobert A. J., Coleridge-Smith P. D., Barr H., Bown S. G. (1987). Photodynamic therapy with phthalocyanine sensitisation: quantitative studies in a transplantable rat fibrosarcoma.. Br J Cancer.

[OCR_00827] Tralau C. J., Young A. R., Walker N. P., Vernon D. I., MacRobert A. J., Brown S. B., Bown S. G. (1989). Mouse skin photosensitivity with dihaematoporphyrin ether (DHE) and aluminium sulphonated phthalocyanine (AlSPc): a comparative study.. Photochem Photobiol.

[OCR_00846] West C. M. (1989). Size-dependent resistance of human tumour spheroids to photodynamic treatment.. Br J Cancer.

[OCR_00850] West C. M., West D. C., Kumar S., Moore J. V. (1990). A comparison of the sensitivity to photodynamic treatment of endothelial and tumour cells in different proliferative states.. Int J Radiat Biol.

